# Postoperative Evaluation of the Quality of Life, Depression, and Anxiety of Temporal Lobe Epilepsy Cohort: A Single Institute Experience in Indonesia

**DOI:** 10.3389/fneur.2021.708064

**Published:** 2021-09-14

**Authors:** Yuriz Bakhtiar, Surya Pratama Brilliantika, Jacob Bunyamin, Muhammad Thohar Arifin, Hardian Hardian, Aris Catur Bintoro, Zainal Muttaqin

**Affiliations:** ^1^Department of Neurosurgery, Faculty of Medicine Diponegoro University/Dr Kariadi Hospital, Semarang, Indonesia; ^2^Department of Physiology, Faculty of Medicine, Diponegoro University, Semarang, Indonesia; ^3^Department of Neurology, Faculty of Medicine Diponegoro University/Dr Kariadi Hospital, Semarang, Indonesia

**Keywords:** post-surgical quality of life, depression, anxiety, temporal lobe epilepsy, Indonesia

## Abstract

**Background:** Besides seizure control, quality of life (QoL) should be considered as an equally important outcome for epilepsy surgery service providers. The paucity of QoL reports from developing countries has enlarged the representation gap between wealthy countries and countries with fewer resources. In this study, we evaluated postoperative QoL in the Indonesian drug-resistant epilepsy cohort where the epilepsy surgery service faces limited resource availability.

**Methods:** We evaluated the QoL in patients with temporal lobe epilepsy who underwent surgery in our epilepsy surgery center in Semarang, Indonesia, from 2001 until 2015. The follow–up period started in 2018 through 2019. Postoperative QoL, depression, and anxiety were evaluated with self-reporting questionnaires including the Quality of Life in Epilepsy Inventory-31, Beck Depression Inventory-II, and Zung Self-Rating Anxiety Scales.

**Results:** Forty returned questionnaires were included in the analysis (male 25, 62.5%; mean age 27.6 ± 9.05 years). The seizure-free cohort (*n* = 22, 55.0%) reported higher scores in most QoL dimensions particularly adjustment, overall QoL, and seizure worry compared to those with persistent seizures. The overall QoL level was correlated with seizure freedom and surgery type. QoL dimensions were negatively correlated with anxiety and depression levels.

**Conclusions:** Postoperative seizure freedom was a major factor of postoperative QoL level. Besides seizure freedom, anxiety and depression levels were also negatively correlated with QoL levels in the Indonesian population.

## Introduction

Epilepsy is a chronic brain disease caused by various factors and is marked by episodic and temporary dysfunction of the central nervous system as a result of excessive neuronal discharges. Around 40 million people with epilepsy (PwE) live in developing nations where the number is expected to increase because of limited medical treatment, drugs, and poor community stigma (1). In Indonesia, the prevalence of epilepsy is estimated as 0.5–0.6% of the total population, reaching ~1.5 million within the 230 million residents of Indonesia. From that number, around 440,000 people would be diagnosed with drug-resistant epilepsy, and half of them are potential candidates for epilepsy surgeries (2). Surgical intervention for epilepsy has been one of the successful methods for seizure control (3). It is estimated that 52–80% of patients were seizure-free at least for a short time after surgery (4–6).

Quality of life is defined as “a state of complete physical, mental, and social well-being and not merely the absence of disease or infirmity” (7). Besides seizure control, QoL should be considered as an equally important outcome for epilepsy surgery service providers (8). Seizure-associated accidents, co-existing physical constraints, lower education levels, unemployment, and psychological issues such as anxiety, depression, low self-esteem, aggression, and sexual dysfunction have been associated with lower QoL in PwE cohorts (9, 10). Surgery has been reported to improve QoL in PwE compared to anti-epileptic drugs (8, 11). This study aimed to observe the QoL of PwE who underwent surgery in our epilepsy surgical center in Indonesia by self-reporting assessments. It is important as there is a scarcity of reports of postsurgical QoL in PwE in low and middle-income countries (LMICs) (12). A previous study has demonstrated a satisfactory seizure-free rate in the Indonesian population, however the study lacked QoL evaluation (13). Furthermore, we also analyzed the effect between postoperative anxiety and depression on QoL levels.

## Methods

This study has been approved by the Institutional Review Board of Dr. Kariadi General Hospital, Semarang, Indonesia. We included patients diagnosed with temporal lobe epilepsy who underwent surgery in Dr. Kariadi Hospital and SMC Telogorejo Hospital Semarang from 2001 until 2015. Follow-ups were performed from 2018 to 2019. Postoperative evaluation included QoL, depression, and anxiety. All participants were required to self-report their QoL, depression, and anxiety using the Indonesian version of the QoL in Epilepsy Inventory (QOLIE)-31, Beck Depression Inventory-II (BDI-II), and Zung-Self-rating Anxiety Scales (Zung-SAS), respectively (14–16). The QOLIE-31 questionnaire consists of 31 items covering eight dimensions i.e., seizure worry, overall QoL, emotional well-being, energy/fatigue, cognition, medication effect, social function, and adjustment. Higher scores indicate a higher self-reported QoL. The BDI-II is a 21-item questionnaire assessing self-reported major depressive disorder symptoms as listed in the Diagnostic and Statistical Manual of Mental Disorders. The Zung-SAS is a 20-item questionnaire assessing the self-reported presence of clinical anxiety. The Indonesian versions of all questionnaires had been tested for validity and reliability (17–19).

The questionnaires were posted to participants' home addresses, which were then followed up by phone interviews should the mails-outs not be returned, or given answers were incomplete. All participants were required to be a minimum of 18 years old at the time of sampling and gave their consent for this study. Participants self-reporting high scores on BDI-II and/or Zung-SAS (cut off index score = 20 and 45, respectively) were contacted to seek advice from mental health care providers to further explore their symptoms (20, 21).

Only completed answers proceeded to descriptive analysis and hypothetical tests. In the descriptive analysis, categorical data were described with frequency and proportion. Dependent variables included QOLIE-31, Zung-SRAS, and BDI-II scores while independent variables included sex, seizure-free status, duration from onset to surgery, surgery type, histopathological data, imaging pathology, and follow-up period. Seizure freedom was defined as the absence of seizure regardless of anti-epileptic drugs consumption after surgery. Meanwhile, QoL scores were described with mean and standard deviation or median if the data distribution were abnormal. Statistical comparison was performed using student's *t*-test or Mann-Whitney test whenever applicable. The difference was considered significant if *p* < 0.05. Data analysis was performed by IBM SPSS Statistics (IBM, USA).

## Results

From 100 sent questionnaires, 62 participants responded; however only 40 completed questionnaires were included in the analysis. We called the remaining 38 participants, however they did not respond to the calls which were then marked as lost to follow-up. Clinical characteristics of the eligible 40 participants (male = 25, 62.5%; mean age 27.6 ± 9.05 years) were summarized in Table 1. Twelve participants (30.0%) were minors at the time of surgery. The mean follow-up period was 7.10 ± 5.56 years. QOLIE-31 scores, depression score, and anxiety score were compared based on the duration of epilepsy from the onset, age at surgery, sex, resection side, pathology, postoperative seizure freedom, and follow-up period.

**Table 1:** Clinical characteristics of the participants.

**Characteristics**	***n* = 40 (%)**
**Age**
Mean ± SD (min-max)	27.6 ± 9.05 (9.0–52.0)
**Sex**
Male	25 (62.5%)
Female	15 (37.5%)
**Duration of seizure**
<10 years	5 (12.5%)
>10 years	35 (87.5%)
**Surgery side**
Right	18 (45%)
Left	22 (55%)
**Imaging diagnosis**
MTS	21 (52.5%)
Non MTS	19 (47.5%)
**Seizure-free status**
Persistent	18 (45.0 %)
Seizure-free	22 (55.0 %)
With drug	15 (68.2%)
Without drug	7 (31.8%)
**Follow up period**
Mean ± SD (min-max)	7.1 ± 5.56 (1.0–34.0)
<5 yrs	12 (30.0%)
>5 yrs	28 (70.0%)
**Pathology**
Focal cortical dysplasia	19 (47.5%)
Hippocampal sclerosis	21 (52.5%)
**Type of Surgery**
Anterior temporal lobectomy	22 (55.0 %)
Selective amygdalohippocampectomy	18 (45.0 %)

*min, minimum; max, maximum; SD, standard deviation*.

**Figure 1:**
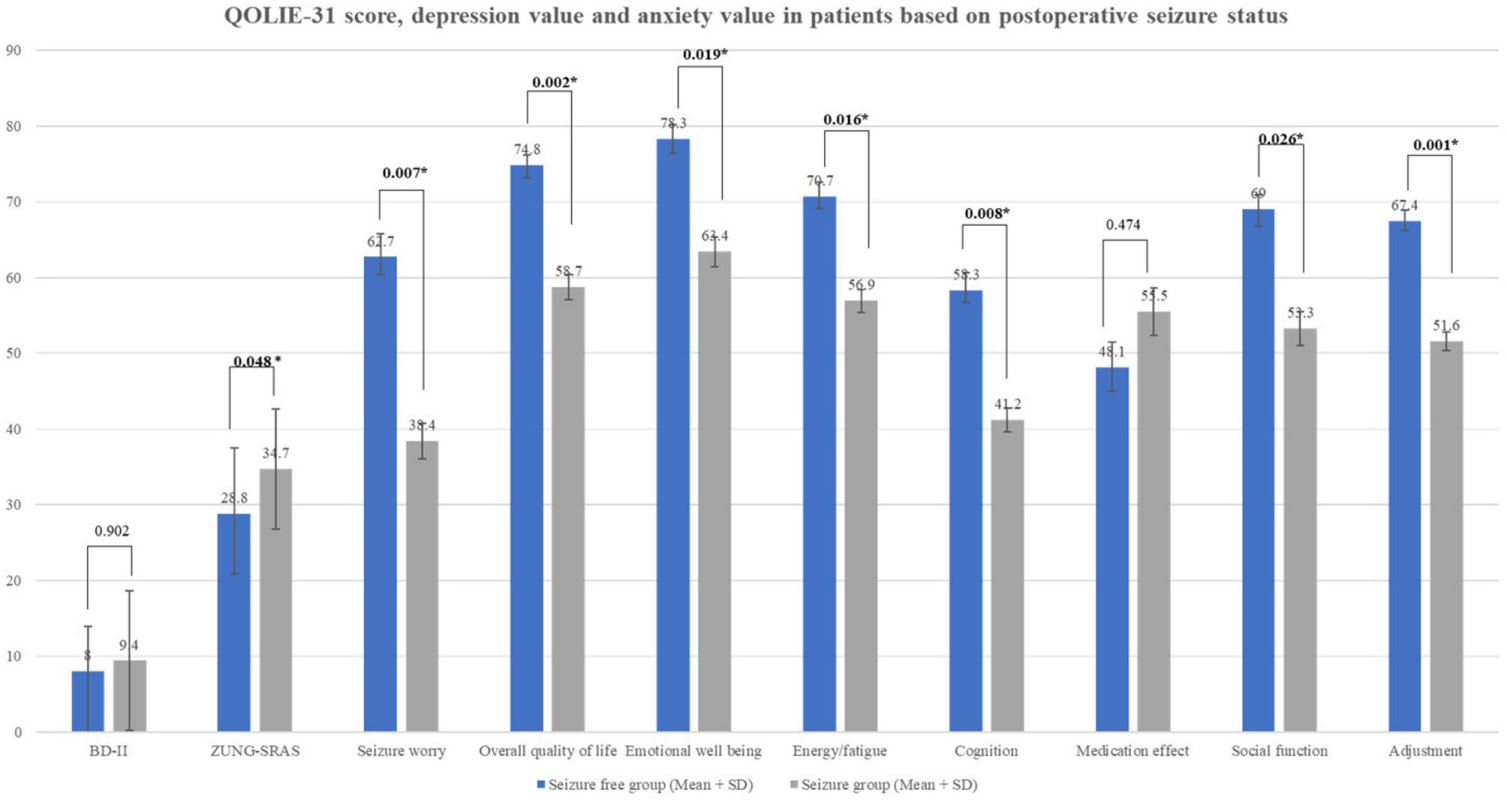
QOLIE-31 score, depression value, and anxiety value in patients based on postoperative seizure status.

Seizure-free subjects had significantly higher QoL dimension scores in many domains including overall quality of life, emotional well-being, energy/fatigue, cognition, social function, adjustment compared to the seizure-persistent group (Figure 1). Among all dimensions, only medication effect was higher in seizure-persistent subjects. The multivariate analysis using two-way ANOVA yielded that the overall quality of life was affected by postoperative seizure status and surgery type (*p* < 0.05, see Table 2). Anxiety level (ZUNG-SRAS score) was significantly lower in the seizure-free cohort and those who were followed-up ≥5 years. On the contrary, depression level was not affected by any demographic and clinical variables.

**Table 2:** Multivariate analysis of BD-II, ZUNG-SRAS, and QOLIE-31 scores based on demographic and clinical variables.

**Evaluation items**		**BD-II**	**ZUNG-SRAS**	**Seizure worry**	**Overall quality of life**	**Emotional well being**	**Energy / fatigue**	**Cognition**	**Medication effect**	**Social function**	**Adjustment**
Sex	Male (*n =* 25)	7.6 ± 1.15 (0.0–19.0)	30.2 ± 1.86 (0.0–45.0)	53.7 ± 3.34 (6.6–100.0)	67.4 ± 1.61 (32.5–87.5)	69.3 ± 1.95 (32.0–100.0)	64.6 ± 1.80 (35.0–100.0)	48.2 ± 1.98 (10.0–80.0)	53.3 ± 2.61 (0.0–100.0)	63.3 ± 1.96 (29.0–100.0)	59.8 ±1.37 (41.31–84.95)
	Female (*n =* 15)	10.1 ± 2.57 (0.0–33.0)	33.9 ± 2.11 (20.0–46.0)	48.4 ± 3.34 (4.0–100.0)	68.0 ± 1.81 (25.0–100.0)	75.6 ± 2.11 (28.0–96.0)	64.3 ±1.98 (30.0–90.0)	54.5 ± 2.43 (15.5–100.0)	48.3 ± 4.18 (0.0–100.0)	59.9 ± 2.62 (6.25–100.0)	61.2 ± 1.89 (28.14–92.95)
***p***		0.417^a^	0.245^b^	0.607^a^	0.855^b^	0.281^b^	0.966^a^	0.406^a^	0.612^b^	0.667^a^	0.645^b^
Seizure duration	<10 years (*n =* 5)	9.0 ± 6.81 (3.0–19.0)	37.8 ± 8.81 (25.0–46.0)	35.2 ± 1.89 (6.6–54.8)	58.5 ±1.68 (40.0–82.5)	55.2 ± 2.06 (28.0–76.0)	56.0 ± 1.59 (35.0–75.0)	46.0 ± 1.87 (15.5−65.83)	41.6 ± 3.17 (0.0–83.3)	56.0 ±1.63 (28.0–66.0)	51.45 ± 1.41 (28.14–62.67)
	≥ 10 years (*n =* 35)	8.6 ± 7.70 (0.0–33.0)	30.6 ± 8.54 (0.0–45.0)	54.1 ±3.02 (4.0–100.0)	68.9 ± 1.65 (25.0–100.0)	74.0 ± 1.92 (32.0- 100.0)	65.7 ± 1.87 (30.0–100.0)	51.3 ±2.21 (10.0–100.0)	52.8 ± 3.27 (0.0–100.0)	62.8 ± 2.32 (6.25–100.0)	61.5 ± 1.56 (38.21–92.95)
***p***		0.821^b^	0.081^b^	0.177^b^	0.249^a^	0.113^a^	0.262^a^	0.590^a^	0.470^b^	0.567^b^	0.357^b^
Imaging diagnosis	MTS (*n =* 21)	9.6 ± 1.85 (0.0–33.0)	31.9 ± 1.60 (20.0–46.0)	54.7 ±6.93 (4.0–100.0)	67.9 ± 3.32 (47.5–100.0)	74.0 ± 4.49 (28.0–100.0)	64.7 ±4.49 (30.0–100.0)	53.9 ± 5.14 (15.5–100.0)	52.3 ±7.04 (0.0–100.0)	59.3 ± 5.47 (6.25–100.0)	61.3 ± 3.89 (28.14–92.95)
	Non MTS (*n =* 19)	7.5 ± 1.44 (0.0–19.0)	31.0 ± 2.37 (0.0–45.0)	48.5 ± 6.30 (6.6–96.0)	67.2 ±4.27 (25.0–87.5)	69.0 ± 4.55 (32.0–100.0)	64.2 ± 3.79 (40.0–100.0)	46.9 ± 4.37 (10.0–80.0)	50.4 ± 7.68 (0.0–100.0)	64.9 ± 4.23 (29.0–100.0)	59.1 ± 3.03 (41.31–84.95)
*p*		0.380^a^	0.957^b^	0.515^a^	0.817^b^	0.444^a^	0.926^a^	0.305^a^	0.853^a^	0.424^a^	0.661^a^
Surgery type	ATL (*n =* 22)	7.7 ± 1.65 (0.0–33.0)	33.3 ± 1.65 (23.0–46.0)	51.6 ± 7.02 (4.0–100.0)	61.7 ± 3.40 (25.0–82.50)	66.3 ± 4.47(28.0–100.0)	57.5 ± 3.64 (30.0–90.0)	45.8 ± 5.27 (10–100.0)	53.7 ± 7.74 (0.0–100.0)	60.8 ± 4.94 (6.25–100.0)	56.4 ± 3.59 (28.14–92.95)
	SelAH (*n =* 18)	9.8 ± 1.71 (0.0–21.0)	29.2 ± 2.27 (0.0–45.0)	51.9 ± 6.11 (15.0–87.0)	74.8 ±3.53 (25.0–87.5)	78.1 ±4.12 (40.0–100.0)	73.0 ± 3.98 (40.0–100.0)	56.4 ±3.69 (31.67–80.0)	48.6 ±6.54 (0.0–100.0)	63.3 ± 5.00 (33.0–100.0)	65.1 ± 3.05 (47.78–89.45)
*p*		0.252^b^	0.270^b^	0.935^b^	**0.011** ^**a**^	0.061^a^	**0.007** ^**a**^	0.110^a^	0.574^b^	0.724^a^	0.507^a^
Resection side	Right (*n =* 18)	7.7 ± 6.85 (0.0–21.0)	31.0 ± 1.04 (0.0–46.0)	53.0 ± 3.5 (4.0–100.0)	68.5 ± 1.27 (47.5–87.5)	68.6 ± 2.03 (28.0–100.0)	59.4 ± 2.04 (35.0–100.0)	49.8 ± 2.74 (10.0–100.0)	53.2 ± 3.06 (0.0–100.0)	62.7 ± 1.86 (28.0–90.0)	59.5 ± 1.74 (28.14–92.95)
	Left (*n =* 22)	9.4 ± 8.08 (0.0–33.0)	31.8 ±7.40 (20.0–45.0)	50.7 ± 2.50 (14.0–87.0)	66.9 ± 1.96 (25.0–100.0)	74.1 ± 2.01 (40.0–100.0)	68.6 ± 1.61 (30–100.0)	51.2 ± 1.58 (24.17–80.0)	50.0 ± 3.45 (0.0–100.0)	61.4 ± 2.49 (6.25–100.0)	61.0 ± 1.44 (38.21–82.09)
*p*		0.576^b^	0.957^b^	0.814^a^	0.767^a^	0.288^b^	0.130^a^	0.854^a^	0.755^a^	0.850^a^	0.771^a^
Histopathological diagnosis	HS (*n =* 19)	9.2 ± 1.81 (0.0–33.0)	32.3 ± 1.58 (20.0–46.0)	54.9 ± 6.61 (4.0–100.0)	67.9 ± 3.16 (47.2–100.0)	74.2 ± 4.29(28.0–100.0)	64.7 ± 4.28 (30.0–100.0)	54.3 ± 4.91 (15.50–100.0)	54.5 ± 7.05 (0.0–100.0)	60.5 ± 5.34 (6.25–100.0)	61.8 ± 3.78 (28.14–92.95)
	CD (*n =* 21)	8.0 ± 1.46 (0.0–19.0)	30.4 ± 2.44 (0.0–450)	47.9 ± 6.63 (4.0–100.0)	67.2 ±4.52 (25.0–87.5)	68.4 ±4.77 (32.0–100.0)	64.1 ± 4.00 (40.0–100.0)	46.0 ±4.52 (10.0–80.0)	47.6 ±7.58 (0.0–100.0)	63.8 ± 4.31 (29–100.0)	58.4 ± 3.12 (41.31–89.45)
*p*		0.882^b^	0.717^b^	0.462^a^	0.757^b^	0.370^a^	0.920^a^	0.232^a^	0.513^a^	0.643^a^	0.507^a^
Seizure status	persisted (*n =* 18)	9.4 ± 9.25 (0.0–33.0)	34.7 ± 7.90 (23.0–46.0)	38.4 ± 2.30 (6.6–80.0)	58.7 ±1.68 (25.0–82.5)	63.4 ± 1.96 (28.0–90.0)	56.9 ± 1.54 (30.0–80.0)	41.2 ± 1.56 (10.0–65.0)	55.5 ± 3.14 (0.0–100.0)	53.3 ± 2.24 (6.25–100.0)	51.6 ± 1.22 (28.14–77.58)
	Free (*n =* 22)	8.0 ± 5.88 (0.0–25.0)	28.8 ± 8.75 (0.0–41.0)	62.7 ± 3.03 (4.0–100.0)	74.8 ±1.30 (47.50–100.0)	78.3 ± 1.83 (32.0–100.0)	70.7 ± 1.88 (40.0–100.0)	58.3 ± 2.29 (10.0–100.0)	48.1 ± 3.36 (0.0–100.0)	69.0 ± 1.94 (39.0–100.0)	67.4 ± 1.47 (47.5–92.95)
*p*		0.902^b^	**0.048** ^**b**^	**0.007** ^**a**^	**0.002** ^**a**^	**0.019** ^**a**^	**0.016** ^**a**^	**0.008** ^**a**^	0.474^a^	**0.026** ^**a**^	**0.001** ^**a**^
Follow-up period	<5 years (*n =* 12)	9.4 ± 9.25 (0.0–33.0)	34.7 ± 7.90 (23.0–46.0)	55.4 ± 3.12 (14.0–100.0)	73.1 ± 1.49 (50.0–100.0)	76.8 ± 1.38 (52.0–96.0)	69.1 ± 1.84 (35.0–90.0)	52.8 ± 1.92 (30.83–100.0)	50.0 ± 3.21 (0.0–100.0)	63.1 ± 1.98 (39.0–100.0)	63.5 ± 1.42 (46.58–92.95)
	≥ 5 years (*n =* 28)	8.0 ± 5.88 (0.0–25.0)	28.8 ± 8.75 (0.0–41.0)	50.2 ± 2.93 (4.0–100.0)	65.3 ±1.71 (25.0–87.5)	69.4 ±2.21 (28.0–100.0)	62.5 ± 1.85 (30.0–100.0)	49.6 ±2.27 (10.0–100.0)	52.0 ±3.31 (0.0–100.0)	61.7 ± 2.37 (6.25–100.0)	58.9 ± 1.62 (28.14–92.45)
*p*		0.902^b^	**0.048** ^**b**^	0.630^a^	0.158^a^	0.686^a^	0.307^a^	0.652^a^	0.858^b^	0.823^a^	0.383^a^

*Mean ± SDV (minimum–maximum);*

a *Unpaired T-test;*

b *Mann-Whitney test. BD-II, Beck Depression Inventory-II; QOLIE-31-P, Quality of Life in Epilepsy Inventory-31-P; ZUNG-SRAS, Zung Self-Rating Anxiety Scales; MTS, mesial temporal sclerosis; ATL, anterior temporal lobectomy; SelAH, selective amygdalohippocampectomy; HS, hippocampal sclerosis; CD, cortical dysplasia. Bold numbers are statistically significant results (p < 0.05)*.

QoL dimensions were negatively correlated with both anxiety and depression levels. We found a negative correlation between medication effect, social function, and adjustment domains with the depression level. Anxiety level was also negatively correlated with QoL as participants with low anxiety scores had higher overall QoL and in emotional well-being, energy/fatigue, cognitive level, and adjustment domains (Table 3).

**Table 3:** Correlation between QOLIE-31 scores and depression and anxiety after surgery.

**QOLIE-31-P score**	**Depression**		**Anxiety**	
	**R**	***p***	**R**	***p***
Seizure worry	−0.098	0.547^a^	−0.269	0.093^a^
Overall quality of life	−0.287	0.072^a^	−0.381	**0.015** ^**a**^
Emotional well-being	−0.209	0.195^a^	−0.608	** <0.001** ^**a**^
Energy/fatigue	−0.243	0.130^a^	−0.548	** <0.001** ^**a**^
Cognition	−0.220	0.172^a^	−0.356	**0.024** ^**a**^
Medication effect	−0.361	**0.022** ^**a**^	−0.646	0.646^a^
Social function	−0.361	**0.022** ^**a**^	−0.207	0.201^a^
Adjustment	−0.337	**0.033** ^**a**^	−0.472	**0.002** ^**a**^

a *Spearman's. QOLIE-31-P, Quality of Life in Epilepsy Inventory-31. Bold indicates significant results (p < 0.05)*.

## Discussion

Considering that the majority of PwE live in developing countries, the paucity of QoL reports from developing countries has enlarged the representation gap between wealthy and low-income countries (22). People with epilepsy in LMICs tend to have a lower QoL level compared to those living in high-income countries (12). In this study, we evaluated postoperative QoL in the Indonesian drug-resistant epilepsy cohort where the epilepsy surgery service faces limited resource availability. Our previous report has demonstrated a satisfactory surgical outcome during 20 years of epilepsy surgery service in Indonesia (13). A report conducted in a non-surgically treated epileptic cohort in Indonesia showed that QoL was correlated with lower education background, increased seizure frequency, anti-epileptic drugs polytherapy, and generalized seizures (23). In general, our findings confirmed that seizure freedom and surgery type affected the overall QoL in our cohort. Moreover, depression and anxiety levels showed a negative correlation with several QoL domains.

Postoperative seizure freedom was a major factor affecting postoperative QoL levels in our cohort. All QoL domains except medication effect were significantly higher in patients with seizure-free status compared to those with persistent seizures. The largest effect was observed on the adjustment and seizure worry domains (*p* = 0.001 and 0.007, respectively). Seizure freedom has been widely reported to alleviate the psychological burden and lead to better performance in social functions (4, 24, 25). Substantial reduction of postoperative AEDs consumption had a beneficial impact on the QoL due to reduced side effects and a reduced healthcare cost (26). Patients with persistent seizures were prone to have worsening postoperative QoL especially if accompanied by a declining memory function (27). Fear of uncontrolled seizures and loss of autonomy were the main factors affecting QoL level as it was inversely proportional to seizure frequency (10, 25). Adequate seizure control was also related to lower depression and anxiety levels among an epilepsy cohort (28). Similarly, we observed that postoperative seizure freedom was related to lower anxiety level (*p* = 0.048), however, there was no effect on depression level. This might be due to the modest sample size and the stigma attached to mental disorders particularly depression, as self-reporting patients might feel uncomfortable in describing their symptoms.

Besides seizure freedom, epilepsy surgery is associated with enhancement of cognitive functions which subsequently had direct and indirect to QoL improvement (29). We observed that patients who underwent selective amygdalohippocampectomy (SelAH) had a higher overall QoL and energy/fatigue domain compared to those who underwent Anterior temporal lobectomy (ATL) (*p* = 0.011 and 0.007, respectively). However, another study described no difference in QoL and depression levels between patients who underwent either SelAH or ATL (30). SelAH is a technique modified from standardized ATL where the amygdala and hippocampus are resected leaving the anterior temporal lobe intact. In our center, SelAH was indicated for patients whose suspected lesion is located in the dominant hemisphere with good preoperative cognitive functions. Compared to unoperated patients, the SelAH cohort had demonstrated a higher QoL level (31). Anterior temporal lobectomy was previously described as improving psychosocial life outcomes including driving, work, independent living, and financial independence (32). However, previous studies had described insignificant differences in neuropsychological outcomes between two procedures (29, 33, 34).

Psychiatric comorbidities are more prevalent in people with epilepsy compared to the general population, particularly the drug-resistant epilepsy cohort (35). We observed a significant correlation between depression and anxiety with QoL scores in several domains, although not all. There were significant correlations between medication effect, social function, and adjustment domains to the depression level (*p* = 0.022, 0.022, and 0.033, *R* = −0.361, −0.361, and −0.337, respectively). Anxiety scores were also negatively correlated with the overall QoL, emotional well-being, energy/fatigue, cognitive level, and adjustment domains (*p* = 0.015, <0.001, <0.001, 0.024, and 0.002, *R* = −0.381, −0.608, −0.548, −0.356, and −0.472, respectively). Depression and anxiety in combination lead to a higher risk of QoL degradation compared to the presence of only a single condition (36). Anxiety and depression symptoms had more effect on QoL compared to seizure frequency, severity, or chronicity (37). Depression level showed a strong negative correlation with QoL particularly in medication effect, social function, and adaptation domains (25, 37, 38). Maladaptive disease perception, a negative social stigma, and medication non-compliance were factors mediating the correlation between depression and QoL in epilepsy patients (39). Increased anxiety level was related to higher seizure frequency and shorter seizure-free duration which was mediated by depressive symptoms (25). Furthermore, this effect extended to caregivers as well, as parents with epileptic children also reported reduced QoL and increased depression and anxiety levels (40).

There were inconsistencies regarding the effect of sex on the QoL level. We found no correlation between sex and QoL level, similar to a prior Korean study (28). On the contrary, another study reported that women participants had higher anxiety levels, while yet another reported that anxiety and depression affected the QoL particularly in men (41, 42). This might occur due to cultural differences, social support, and gender-based norms which are different among cultures. There were also non-remarkable differences in QoL regarding follow-up periods. A study showed that postoperative QoL was improved after 1 year without any significant impact thereafter (43). This could be due to the ceiling effect after the patients have reached the average QoL score of the normal population. Moreover, other clinical characteristics such as epilepsy duration, imaging diagnosis, histopathological diagnosis, and resection side were not significantly correlated with postoperative QoL scores.

The limitations of this study were that we were unable to compare preoperative and postoperative QoL, depression, and anxiety scores due to the unavailability of preoperative scores. Since this is a retrospective study we could not eliminate the possibility of selection bias. We also acknowledge that self-reporting questionnaires might introduce self-report bias. However, this study remains valuable in describing postoperative QoL, anxiety, and depression levels of a drug-resistant epilepsy cohort in the Indonesian population.

## Conclusion

Our findings confirmed that surgery resulted in satisfactory postoperative QoL in the Indonesian cohort. Besides seizure freedom, postoperative QoL should be evaluated in epilepsy surgery candidates particularly in LMICs due to the paucity of reports. Postoperative seizure freedom was a major factor affecting QoL and anxiety levels besides surgery type. Almost all QoL domains were higher in the seizure-free group compared to the seizure-persistent one. Anxiety and depression levels negatively correlated with QoL levels in the Indonesian population. This should raise awareness regarding the importance of postoperative QoL evaluation, particularly in underreported populations.

## Data Availability Statement

The raw data supporting the conclusions of this article will be made available by the authors, without undue reservation.

## Ethics Statement

The studies involving human participants were reviewed and approved by Institutional Review Board Faculty of Medicine Diponegoro University, Semarang, Indonesia. The patients/participants provided their written informed consent to participate in this study.

## Author Contributions

All authors listed have made a substantial, direct and intellectual contribution to the work, and approved it for publication.

## Conflict of Interest

The authors declare that the research was conducted in the absence of any commercial or financial relationships that could be construed as a potential conflict of interest.

## Publisher's Note

All claims expressed in this article are solely those of the authors and do not necessarily represent those of their affiliated organizations, or those of the publisher, the editors and the reviewers. Any product that may be evaluated in this article, or claim that may be made by its manufacturer, is not guaranteed or endorsed by the publisher.

## References

[1] Asadi-pooya AA Torabinami M. Knowledge and attitude towards epilpesy among biology teachers in Fars Province, Iran. Iran J Child Neurol. (2012) 6:13–18. 10.22037/ijcn.v6i1.2926

[2] Muttaqin Z. Epilepsy surgery in Indonesia: achieving a better result with limited resources. Bali Med J. (2012) 1:57–63.

[3] Wiebe S Blume WT Girvin JP Eliasziw M. A randomized, controlled trial of surgery for temporal-lobe epilepsy. N Engl J Med. (2001) 345:311–8. 10.1056/NEJM200108023450501 11484687

[4] Tanriverdi T Poulin N Olivier A. Life 12 years after temporal lobe epilepsy surgery: a long-term, prospective clinical study. Seizure. (2008) 17:339–49. 10.1016/j.seizure.2007.11.003 18083604

[5] Alonso NB Azevedo AM Centeno RS Ferreira Guilhoto LMF Ferreira Caboclo LOS Yacubian EMT. Employment and quality of life in mesial temporal lobe epilepsy with hippocampal sclerosis: is there a change after surgical treatment? J Epilepsy Clin Neurophysiol. (2009) 15:89–93. 10.1590/S1676-26492009000200008

[6] De Tisi J Bell GS Peacock JL McEvoy AW Harkness WF Sander JW. The long-term outcome of adult epilepsy surgery, patterns of seizure remission, and relapse: a cohort study. Lancet. (2011) 378:1388–95. 10.1016/S0140-6736(11)60890-8 22000136

[7] World Health Organization WHOQOL : Measuring Quality of Life. World Health Organization. Division of Mental Health and Prevention of Substance Abuse. Available online at: https://apps.who.int/iris/handle/10665/63482 (1997).

[8] Lotfinia M Maloumeh EN Asaadi S Omidbeigi M Sharifi G Asadi B. Health-related quality of life after epilepsy surgery: a prospective, controlled follow-up on the Iranian population. Sci Rep. (2019) 9:1–11. 10.1038/s41598-019-44442-6 31133687 PMC6536509

[9] Loring DW Meador KJ Lee GP. Determinants of quality of life in epilepsy. Epilepsy Behav. (2004) 5:976–80. 10.1016/j.yebeh.2004.08.019 15582847

[10] Izci F Findikli E Camkurt MA Tuncel D Sahin M. Impact of aggression, depression, and anxiety levels on quality of life in epilepsy patients. Neuropsychiatr Dis Treat. (2016) 12:2595–603. 10.2147/NDT.S113041 27785037 PMC5067059

[11] Fiest KM Sajobi TT Wiebe S. Epilepsy surgery and meaningful improvements in quality of life: results from a randomized controlled trial. Epilepsia. (2014) 55:886–92. 10.1111/epi.12625 24735200

[12] Saadi A Patenaude B Mateen FJ. Quality of life in epilepsy-−31 inventory (QOLIE-31) scores: a global comparison. Epilepsy Behav. (2016) 65:13–7. 10.1016/j.yebeh.2016.09.032 27838562

[13] Arifin MT Hanaya R Bakhtiar Y Bintoro AC Iida K Kurisu K. Initiating an epilepsy surgery program with limited resources in Indonesia. Sci Rep. (2021) 11:1–12. 10.1038/s41598-021-84404-5 33658553 PMC7930083

[14] Zung W. A rating instrument for anxiety disorders. Psychosomatics. (1971) 12:371–9. 10.1016/S0033-3182(71)71479-0 5172928

[15] Beck AT Steer RA Brown G. Manual for the Beck Depression Psychological, Inventory. 2nd ed (BDI-II). San Antonio, TX: Psychological Corporation (1996).

[16] Cramer JA Perrine K Devinsky O Bryant-Comstock L Meador F Hermann B. Development and cross-cultural translations of a 3 1-item quality of life in epilepsy inventory. Epilepsia. (1998) 39:81–8. 10.1111/j.1528-1157.1998.tb01278.x 9578017

[17] Setyowati A Chung M Yusuf A. Development of self report assessment tool for anxiety among adlolescents: Indonesian version of the zung self rating anxiety scale. J Public Health Africa. (2010) 54:107–11. 10.4081/jphia.2019.1172

[18] Gunadharma S Nurimaba N Rochayatin O. Validation and Reliability Test of the Indonesian Version of QOLIE-31. Neurona. (2015) 32.

[19] Uin S Hidayatullah S. Uji validitas konstruk Beck depression inventory-II (BDI-II). J Pengukuran Psikol dan Pendidik Indones. (2018) 4:1–13. 10.15408/jp3i.v4i1.9259

[20] Kapci EG Uslu R Turkcapar H Karaoglan A. Beck depression inventory II: evaluation of the psychometric properties and cut-off points in a Turkish adult population. Depress Anxiety. (2008) 25:104–10. 10.1002/da.20371 17876817

[21] Dunstan DA Scott N. Assigning clinical significance and symptom severity using the zung scales: levels of misclassification arising from confusion between index and raw scores. Depress Res Treat. (2018) 2018: 9250972. 10.1155/2018/9250972 29610683 PMC5828114

[22] Beghi E. The epidemiology of epilepsy. Neuroepidemiology. (2020) 54:185–91. 10.1159/000503831 31852003

[23] Hawari I Syeban Z Lumempouw SF. Low education, more frequent of seizure, more types of therapy, and generalized seizure type decreased quality of life among epileptic patients. Med J Indones. (2007) 16:101–3. 10.13181/mji.v16i2.265

[24] Kessler RC Lane MC Shahly V Stang PE. Accounting for comorbidity in assessing the burden of epilepsy among US adults: results from the National Comorbidity Survey Replication (NCS-R). Mol Psychiatry. (2012) 17:748–58. 10.1038/mp.2011.56 21577213 PMC3165095

[25] Johnson AL McLeish AC Alsaid-Habia T Shear PK Privitera M. Anxiety sensitivity as a predictor of epilepsy-related quality of life and illness severity among adult epilepsy. Cognit Ther Res. (2019) 43:6–13. 10.1007/s10608-018-9951-4

[26] Acar G Acar F Gedik B Yilmaz A Altug F Aras D. Quality of life and cost analysis following epilepsy surgery in turkish patients. J Turkish Epilepsi Soc. (2011) 17:39–45. 10.5505/epilepsi.2011.72792

[27] Langfitt JT Westerveld M Hamberger MJ Walczak TS Cicchetti DV Berg AT. Worsening of quality of life after epilepsy surgery. Neurology. (2007) 68:1988. 10.1212/01.wnl.0000264000.11511.30 17548548

[28] Lee SJ Kim JE Seo JG Cho YW Lee JJ Moon HJ. Predictors of quality of life and their interrelations in Korean people with epilepsy: a MEPSY study. Seizure. (2014) 23:762–8. 10.1016/j.seizure.2014.06.007 25008245

[29] Gül G Yandim Kuşcu D Özerden M Kandemir M Eren F. Tugcu B. Cognitive outcome after surgery in patients with mesial temporal lobe epilepsy. Noropsikiyatri Ars. (2017) 54:43–48. 10.5152/npa.2016.13802 28566958 PMC5439471

[30] Wendling AS Hirsch E Wisniewski I Davanture C Ofer I Zentner J. Selective amygdalohippocampectomy versus standard temporal lobectomy in patients with mesial temporal lobe epilepsy and unilateral hippocampal sclerosis. Epilepsy Res. (2013) 104:94–104. 10.1016/j.eplepsyres.2012.09.007 23022178

[31] Aydemir N Özkara Ç Canbeyli R Tekcan A. Changes in quality of life and self-perspective related to surgery in patients with temporal lobe epilepsy. Epilepsy Behav. (2004) 5:735–42. 10.1016/j.yebeh.2004.06.022 15380127

[32] Jones J Blocher J Jackson D. Life outcomes of anterior temporal lobectomy: serial longterm follow-up evaluations. Neurosurgery. (2014) 73:1018–25. 10.1227/NEU.0000000000000145 24056319 PMC4162125

[33] Hu WH Zhang C Zhang K Meng FG Chen N Zhang JG. Selective amygdalohippocampectomy versus anterior temporal lobectomy in the management of mesial temporal lobe epilepsy: a meta-analysis of comparative studies A systematic review. J Neurosurg. (2013) 119:1089–97. 10.3171/2013.8.JNS121854 24032705

[34] Josephson CB Dykeman J Fiest KM Liu X Sadler RM Jette N. Systematic review and meta-analysis of standard vs selective temporal lobe epilepsy surgery. Neurology. (2013) 80:1669–76. 10.1212/WNL.0b013e3182904f82 23553475

[35] Tellez-Zenteno JF Patten SB Jetté N Williams J Wiebe S. Psychiatric comorbidity in epilepsy: a population-based analysis. Epilepsia. (2007) 48:2336–44. 10.1111/j.1528-1167.2007.01222.x 17662062

[36] Kwon OY Park SP. Frequency of affective symptoms and their psychosocial impact in Korean people with epilepsy: a survey at two tertiary care hospitals. Epilepsy Behav. (2013) 26:51–6. 10.1016/j.yebeh.2012.10.020 23207517

[37] Johnson EK Jones JE Seidenberg M Hermann BP. The relative impact of anxiety, depression, and clinical seizure features on health-related quality of life in epilepsy. Epilepsia. (2004) 45:544–50. 10.1111/j.0013-9580.2004.47003.x 15101836

[38] Cramer JA Brandenburg N Xu X. Differentiating anxiety and depression symptoms in patients with partial epilepsy. Epilepsy Behav. (2005) 6:563–9. 10.1016/j.yebeh.2005.02.017 15907750

[39] Alsaadi T Kassie S El Hammasi K Shahrour TM Shakra M Turkawi L. Potential factors impacting health-related quality of life among patients with epilepsy: results from the United Arab Emirates. Seizure. (2017) 53:13–7. 10.1016/j.seizure.2017.10.017 29096164

[40] Lv R Wu L Jin L Lu Q Wang M Qu Y. Depression, anxiety and quality of life in parents of children with epilepsy. Acta Neurol Scand. (2009) 120:335–41. 10.1111/j.1600-0404.2009.01184.x 19456304

[41] Pintor L Bailles E Fernández-Egea E Sánchez-Gistau V Torres X Carreño M. Psychiatric disorders in temporal lobe epilepsy patients over the first year after surgical treatment. Seizure. (2007) 16:218–25. 10.1016/j.seizure.2006.12.004 17204436

[42] Yue L Yu P Zhao D Wu D Zhu G Wu X. Determinants of quality of life in people with epilepsy and their gender differences. Epilepsy Behav. (2011) 22:692–6. 10.1016/j.yebeh.2011.08.022 21964448

[43] Dias LA Angelis G de Teixeira WA Casulari LA. Long-Term Seizure, Quality of Life, Depression, and Verbal Memory Outcomes in a Controlled Mesial Temporal Lobe Epilepsy Surgical Series Using Portuguese-Validated Instruments. World Neurosurg. (2017) 104:411–417. 10.1016/j.wneu.2017.05.004 28502691

